# Clinical Reversible Myelopathy in T-Cell Lymphoblastic Lymphoma Treated with Nelarabine and Radiotherapy: Report of a Case and Review of Literature of an Increasing Complication

**DOI:** 10.4084/MJHID.2015.025

**Published:** 2015-03-01

**Authors:** Maria Chiara Tisi, Giuseppe Ausoni, Maria Gabriella Vita, Tommaso Tartaglione, Mario Balducci, Luca Laurenti, Patrizia Chiusolo, Stefan Hohaus, Simona Sica

**Affiliations:** 1 Institute of Hematology, Catholic University S. Cuore, Rome; 2 Institute of Neurology, Catholic University S. Cuore, Rome; 3 Institute of Radiology, Catholic University S. Cuore, Rome; 4 Institute of Radiation Oncology, Catholic University S. Cuore, Rome

## Abstract

Eleven cases of neurological defects in T-ALL patients treated with nelarabine have been described in the last 4 years, seven of these after stem cell transplantation (SCT) for T Lymphoblastic Lymphoma (T-LBL). Most of these patients had an unfavorable outcome or irreversible neurological damage. We now report the case of a 41-year-old woman suffering from T-LBL who presented with severe, but reversible myelopathy after receiving nelarabine-based treatment and mediastinal radiotherapy, and we provide a review of the literature on the topic.

## Introduction

Clinical responses to Nelarabine have been demonstrated in various T-cell malignancies, but neuropathy is the most predominant adverse effect associated with this drug.^1^ The vast majority of nelarabine-related toxicity cases described in the last 4 years also received radiotherapy as part of the planned treatment or the conditioning regimen. Most of these patients had an unfavorable outcome or irreversible neurological damage. Herein we describe the case of a 41-year-old woman suffering from T Lymphoblastic Lymphoma, who presented with severe, but reversible myelopathy after treatment with nelarabine and radiotherapy.

## Case Report

A 41-year-old woman with a medical history of thyroiditis presented at our Institution with a mediastinal mass up to 9 centimeters in diameter, without involvement of other organs or lymph nodes (LN). A biopsy of the mass was performed, and a diagnosis of T Lymphoblastic Lymphoma was established (Ki67 90%). The peripheral white blood cell (WBC) count was normal, and a bone marrow biopsy was inconclusive. No involvement of the central nervous system (CNS) was detected. Chemotherapy according to the GMALL protocol^2^ was started, including intrathecal CNS prophylaxis with Methotrexate alternated with Cytarabine. A complete remission (CR) by conventional criteria^3^ was achieved after two cycles of induction chemotherapy, although a residual infiltrate of T lymphocytes (6%) was documented in the bone marrow biopsy. The patient then underwent mediastinal (2400 cGy), and cranial radiotherapy (2400 cGy) followed by consolidation with HDAC/MITOX and HDMTX/ASP.^2^ During chemotherapy, major adverse effects were gastrointestinal symptoms caused by a documented cytomegalovirus colitis. The planned treatment was stopped ahead of schedule because the patient was not considered in CR due to residual disease in the bone marrow. In order to enhance the response in preparation for allogeneic stem cell transplantation, she was then given nelarabine (two cycles of 1500 mg/square meter on days 1,3 and 5 of a 21-day cycle).

**Figure 1:**
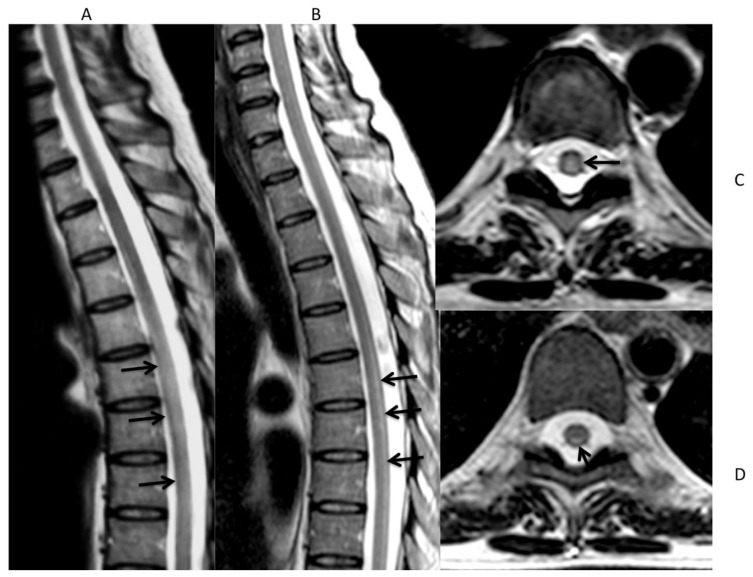
**a,b,c,d.** Sagittal and axial FSE T2w images at the onset of symptoms (a,c) and after 5 months (b,d). MR images obtained at the onset of symptoms show a diffuse hyperintensity of the dorsal spinal cord gray matter (arrows in a and c). After 5 months MRI shows only a linear residual hyperintensity involving the posterior spinal cord gray matter (arrows in b and d)

One month after the last dose of nelarabine, she was submitted to an unrelated matched hematopoietic stem cell transplant. During the conditioning regimen with busulfan and cyclophosphamide, she developed progressive sensory loss in the lower limbs, paraparesis, and ataxia, (grade 3 toxicity according to NCI-CTCAE v4.03).^4^ In addition, she complained of urinary retention that required bladder catheterization. Treatment was continued, and she received hematopoietic stem cells peripheral blood G-CSF mobilized; cyclosporine, rabbit anti-thymocyte globulin and MTX were administered as GVHD prophylaxis. Spinal Magnetic Resonance Imaging (MRI) with gadolinium revealed a hyperintense T2w signal from vertebral level D5 to D11, consistent with inflammatory myelitis (Figure 1a,c). A lumbar puncture was performed that was negative for both leukemic and/or infectious CNS involvement. The patient received steroid therapy with dexamethasone 4 mg twice daily for 15 days. Later, when the patient recovered from aplasia, intensive rehabilitation physical therapy was started, with progressive improvement. The last MRI performed 5 months later (Figure 1b,d) showed the persistence of spinal cord alteration. At the moment of writing this report, 22 months after the initial damage, the patient is in complete remission and able to walk with a mobility aid (5/6 according to ADL-Activities of Daily Living score).

## Discussion

A frequent major dose-limiting side effect of many chemotherapeutics agents, including vinca alkaloids, taxanes, thalidomide and newer agents such as bortezomib, is peripheral neuropathy. The incidence and degree of neuropathy depends on the type of cytotoxic drug, the duration of administration, the cumulative dose, and pre-existing peripheral neuropathy. The damage is, in many cases, only partially reversible, and sometimes even completely irreversible. In this study, we report the case of 41-year-old woman suffering from severe myelopathy after nelarabine treatment, mediastinal radiotherapy and allogeneic stem cell transplantation for T-LBL.

Nelarabine is a nucleoside pro-drug of 9-beta-D-arabinofuranosyl guanine (ara-G). It was approved in October 2005 for the treatment of pediatric and adult patients diagnosed with T-cell acute lymphoblastic leukemia (T-ALL) and T-cell lymphoblastic lymphoma (T-LBL), refractory or relapsed after treatment with at least two chemotherapeutic regimens.^5^ Clinical responses to nelarabine have been demonstrated in various T-cell malignancies, but neuropathy is the most predominant adverse effect associated with this drug. The incidence of neuropathy correlates with the dose administered. The reported neurological symptoms occurs around the 12th day after the beginning of treatment; they are often preceded by transient somnolence, malaise, and overt fatigue, occurring 6 to 8 days after the initiation of nelarabine treatment.^5^ The patient described in our report developed a severe myelopathy with sensory loss, paraparesis, ataxia and sphincteric dysfunction. Since leukemic infiltration and ischemic, hemorrhagic or infectious etiology were ruled out, the myelopathy was attributed to cumulative drug toxicity from nelarabine and the damage caused on the spinal cord to the mediastinal radiotherapy.

The neurological dose-limiting toxicity of nelarabine was initially described in a phase I study by Kurtzberg et al., where 72% of patients enrolled experienced a neurological event.^6^ Substantial neurological toxicity was also observed in a phase II study by Berg et al., who described a grade ≥3 neurological event in 18% of patients.^7^ DeAngelo et al. reported 39 refractory or relapsed T-ALL and T-LBL in adults treated with nelarabine as single-agent: the drug showed a substantial activity, with a complete remission rate of 31% and an overall response rate of 41%. In this study there was only one grade 4 adverse event of the nervous system.^8^

To date, eleven cases of irreversible neurological defects in T-ALL patients treated with nelarabine have been described in the last 4 years,^9^–^14^ seven of these after stem cell transplantation (SCT) for T-LBL.^10^,^11^ Detailed clinical information on these previously reported cases are summarized in Table 1. Patients received nelarabine either prior to SCT or after SCT for lymphoma progression. The vast majority also received radiotherapy as part of the planned treatment or in the conditioning regimen. In the report from Kawakami et al, an excess of nelarabine neurotoxicity (up to 50%) was detected after HLA-haploidentical SCT.^11^ In the recent paper from Ngo et al, concurrent administration of single dose intrathecal cytosine arabinoside was felt to exert an additive neurotoxic effect due to the close timing of administration to nelarabine.^14^ MRI findings, when reported, are superimposable resulting in T2-weighted and FLAIR hyperintensity predominantly at thoracic or cervical level. In conclusion, we emphasize that the onset of not specific symptoms, like “symmetric neurologic symptoms”, seldom reversible despite intensive rehabilitation, should raise the suspicion for nelarabine toxicity in patients who received a previous treatment with this active drug usually after a short latency period, particularly if combined with radiotherapy or intrathecal administration of cytotoxic drug.

## Figures and Tables

**Table 1:** Cases of neurological defects in T-ALL patients treated with nelarabine previously reported in the literature.

Case/Reference	*Papayannidis C,* [9] *Am J Hematol 2010*	*Forcade E,* [10] *Biol Blood Marrow* *Transplant 2013*	*Kawakami M,* [11] *Am J Hematol 2013*	*Gollard R,* [12] *J Clin Oncol 2013*	*Hartz B,* [13] *Am J Hematol 2013*	*Ngo D,* [14] *J Oncol Pharm Practice*	*This report*
**N of patients**	1	4/11	3/6	1	1	1	1
**Diagnosis**	ALL-T	ALL-T	ALL-T	ALL-T	ALL-T	T lymphoblastic lymphoma	T lymphoblastic lymphoma
**Previous treatment**	Standard CHT/Nelarabine	Standard CHT/allo-BMT Nelarabine after allo-BMT	Standard CHT/allo-BMT Nelarabine after allo-BMT (1/3) Radiotherapy 1/3	Standard CHT/Nelarabine	Standard CHT/Nelarabine	Standard CHT/autologous-BMT Nelarabine before allo-BMT+ it cytarabine	Standard CHT/allo-BMT Nelarabine before allo-BMT Radiotherapy
**CNS involvement**	no	2 patients at relapse	no	no	no	no	no
**Conditioning regimen**	Transplant not performed	Myeloablative(TBI)/RIC (TBI unspecified )	n1 RIC-BCNU n2 MAC including TBI n3 MAC including TBI	Transplant not performed	Transplant not performed	RIC-fludarabine+TBI (200 cGy)	Myeloablative(Busulfan/Cyclophosphamide)
**Neurological symptoms**	Paresthesias in lower limbs, defect in equilibrium and walking impairment Sphincteric dysfunctions	1pt Dysautonomia (G1) 1pt Paresthesia (G1) 1pt Peripheral sensory neuropathy (G2) 1pt Ataxia/peripheral sensory neuropathy (G2/3)	n1 Paresthesias and muscle weakness in lower limbs; urinary dysfunction n2 Muscle weakness and walking impairment n3 generalized paresthesia and muscle weakness in lower extermities, walking impairement	Left foot drop, paraplegia, weakness of upper extremities evolving to complete flaccid paralysis, ataxia Urinary retention	Seizures, Guillan-Barré-like syndrome; loss of sensitivity and reflexes of the lower limbs, dysesthesia of the arms and loss of motor control.	Bilateral lower extremity numbness, gait instability; urinary incontinence	Progressive sensory loss in lower limbs, paraparesis, ataxia. Urinary retention
**MRI findings**	Spinal cord alterations consistent with transverse acute myelitis T5	Not described	N1 and n2 (n3 not described) T2-weighted and FLAIR hyperintensity of spinal cord (cervical and thoracic)	T2-weighted and FLAIR hyperintensity at vertebral level T6-T12	T2-weighted hyperintensity of spinal cord; T2 signal changes of cranial nerves (restricted water diffusion, cytotoxic edema)	Increased T2 signal within the dorsal columns from C2 to C6 consistent with subacute combined generation	Hyperintense T2w signal from vertebral level T5 to T11, consistent with inflammatory myelitis
**Treatment**	Intravenous corticosteroids	Not described	Intravenous immunoglobulin	Dexamethasone, cyanocobalamin, folate and multivitamin	Not described	Intensive rehabilitation physiotherapy	Dexamethasone 4 mg twice daily dosing for 15 days; Intensive rehabilitation physiotherapy
**Outcome**	Irreversible complete paraplegia	Not described	Death (n1 and n2 progression of lymphoma/n3 gastrointestinal hemorrhage: GVHD)	Death (recurrence of leukemia)	Death (blast crisis)	Partial recovery from damage; progression of lymphoma (palliative radiotherapy)	Recovery from damage; CR at 16 months after transplantation

**Abbreviations:** N_number; CNS_Central Nervous System; MRI_ Spinal Magnetic Resonance; ALL-T_ T-cell acute lymphoblastic leukemia; CHT_Chemotherapy; allo-BMT_allogenic Bone Marrow Transplantation; TBI_Total Body Irradiation; RIC_Reduced Intensity Conditioning; pt_patient; G_grade; BCNU_carmustine; n_number; MAC_Myeloablative Conditioning; it_intrathecal; CR_Complete Remission.
